# Lactate: an intracellular metabolite regulates cell cycle progression

**DOI:** 10.1093/lifemeta/load017

**Published:** 2023-04-24

**Authors:** Jinke Cheng, Edward T H Yeh

**Affiliations:** Department of Biochemistry and Molecular Cell Biology, Shanghai Key Laboratory for Tumor Microenvironment and Inflammation, Shanghai Jiao Tong University School of Medicine, Shanghai 200025, China; Department of Internal Medicine, University of Arkansas for Medical Sciences, Little Rock, AR 72205, United States

**The Chouchani lab recently reported in *Nature* that a dynamic intracellular lactate is a physiological regulator for cell cycle progress. They also showed that accumulated lactate in cell mitosis directly binds and inhibits Sentrin/SUMO-specific protease 1 to enrich SUMO2/3-modification of anaphase-promoting complex 4 (APC4), which promotes the degradation of APC/C complexes, leading to mitosis exit.**

Cellular metabolism is an important driver of cellular activities [1]. On the one hand, cellular metabolism adjusts to meet the needs of cellular activity [1]. For example, in proliferating cells, aerobic glycolytic metabolism increases, and the intermediate products of glycolytic metabolism are used as building blocks for synthesis of macromolecules needed for cell proliferation. On the other hand, the metabolites produced by cellular metabolism regulate cell activity. The accumulated studies demonstrate that intracellular metabolites are mostly used as donors to modify biological macromolecules, thus regulating cell activity [2]. For example, acetyl-CoA is not only a metabolite of glucose and fatty acid metabolism, but also a donor of acetyl group for protein acetylation. Similarly, acyl groups for succinylation, crotonylation, lactylation, and other protein acylation modifications are produced by metabolic processes such as tricarboxylic acid cycle, amino acid metabolism, fatty acid β oxidation, glycolysis, and other metabolic processes [2]. Methyl donors modified with DNA, RNA, and protein methylation are provided by one-carbon metabolism.

However, not much is known whether metabolites regulate protein activity in other ways [3]. The Chouchani lab [4] found that lactate, the terminal product of anerobic glycolytic metabolism, binds and inhibits Sentrin/SUMO-specific protease 1 (SENP1) by forming a complex with zinc in the SENP1 active site. They further showed that the catalytic domain of the SENP1 enzyme has a zinc-binding pocket, which binds zinc to inhibit its enzymatic activity. Lactate exerts its inhibitory activity by binding to zinc and stabilizes the binding of zinc/lactate to the zinc-binding pocket. As consequence of the reduced SENP1 activity, SUMO2/3-modification of anaphase-promoting complex 4 (APC4) is accumulated, promoting degradation of APC/C complexes, and mitosis exit. This study illustrates a new model that metabolites produced by cellular metabolism can directly bind and modulate protein function.

Cell proliferation requires a large amount of macromolecular building blocks [1]. In different phases of the cell cycle, the need for macromolecules is different. For example, in the S phase, DNA replication requires a large amount of nucleic acids, thus glycolytic metabolism will increase significantly to provide building blocks for nucleic acid synthesis. Therefore, a large amount of lactic acid will be produced in the cells at S phase. Lactate production rates are associated with cell proliferation, suggesting that lactate may be a regulator to modulate the process of cell proliferation. However, how lactate affects cell proliferation is unknown. The authors showed that the intracellular lactate concentration increased from 6 to 15–20 mmol/L when cells entered the mitosis phase. The accumulated lactate became an important regulator of cell division by directly inhibiting SENP1 deSUMOylation activity, which caused an accumulation of SUMO2/3-modification of APC4, promoting the degradation of the APC/C complexes.

SUMOylation is a reversible process regulated by SUMO E1/E2/E3 and the SENP family or proteases [5]. The authors focused their analysis on the effect of zinc and lactate on SENP1. They also showed that zinc and lactate have a combined suppressive effect on SENP5. There are still some questions to be resolved: could SENP5 also play a role in cell division? Moreover, the authors did not rule out that lactate may also affect SUMO E1, E2, or E3. SENP1 has been shown to play a critical role in the regulation of hypoxia-inducible factor 1 alpha stability [6]. Could lactate modulate the hypoxic response through inhibition of SENP1? SENP1 is also important for the regulation of insulin secretion [7]. Could inhibition of SENP1 by lactate play an important pathophysiological role in insulin secretion during sepsis?
